# A Novel Cryptic Binding Motif, LRSKSRSFQVSDEQY, in the C-Terminal Fragment of MMP-3/7-Cleaved Osteopontin as a Novel Ligand for α9β1 Integrin Is Involved in the Anti-Type II Collagen Antibody-Induced Arthritis

**DOI:** 10.1371/journal.pone.0116210

**Published:** 2014-12-29

**Authors:** Shigeyuki Kon, Yosuke Nakayama, Naoki Matsumoto, Koyu Ito, Masashi Kanayama, Chiemi Kimura, Hitomi Kouro, Dai Ashitomi, Tadashi Matsuda, Toshimitsu Uede

**Affiliations:** 1 Division of Molecular Immunology, Institute for Genetic Medicine, Hokkaido University, Sapporo, Hokkaido, Japan; 2 Department of Immunology, Faculty of Pharmaceutical Sciences, Hokkaido University, Sapporo, Hokkaido, Japan; Institute of Hepatology, Foundation for Liver Research, United Kingdom

## Abstract

Osteopontin (OPN) is a multifunctional protein that has been linked to various intractable inflammatory diseases. One way by which OPN induces inflammation is the production of various functional fragments by enzyme cleavage. It has been well appreciated that OPN is cleaved by thrombin, and/or matrix metalloproteinase-3 and -7 (MMP-3/7). Although the function of thrombin-cleaved OPN is well characterized, little is known about the function of MMP-3/7-cleaved OPN. In this study, we found a novel motif, LRSKSRSFQVSDEQY, in the C-terminal fragment of MMP-3/7-cleaved mouse OPN binds to α9β1 integrin. Importantly, this novel motif is involved in the development of anti-type II collagen antibody-induced arthritis (CAIA). This study provides the first *in vitro* and *in vivo* evidence that OPN cleavage by MMP-3/7 is an important regulatory mechanism for CAIA.

## Introduction

Osteopontin (OPN) is a multifunctional protein and has been linked to many physiological and pathological events, such as cell migration, cell survival, intractable inflammations and tumor metastasis [1]–[3]. OPN exerts its functions via binding integrin receptors. OPN has two integrin binding domains, RGD and SVVYGLR sequences [2], [4]. OPN binds to RGD-recognizing integrins such as α5β1 and αvβ3 integrins via the RGD domain [2], [5], [6]; and binds to α9β1 and α4β1 integrins via the SVVYGLR domain [4], [7], [8]. OPN undergoes post-translational processing by thrombin [9], transglutaminase 2 (TG2) [10], enteropeptidase [11], carboxypeptidase B [12], or matrix metalloproteinase-3 (stromelysin-1) and -7 (matrilysin) [13]. Thrombin-cleaved OPN acquires some new functions, one of which is binding to α9β1 integrin via a cryptic binding sequence, SVVYGLR (SLAYGLR in mice) [4], [14], [15], but full-length OPN does not bind to α9β1 integrin [14]. Another function is promotion of adhesion and spreading [15]–[18]. OPN polymerized by TG2 gains a novel function, which is neutrophil chemotaxis mediated by α9β1 integrin [19], [20]. Thus, the effect and outcome of thrombin cleavage of OPN and polymeric OPN is well characterized. Conversely, little is known about the function and/or outcome of MMP-3 or MMP-7 (MMP-3/7)-cleaved OPN. In the present study, we identified a novel binding motif, ^152^LRSKSRSFQVSDEQY^166^ in the C-terminal fragment of MMP-3/7-cleaved mouse OPN, and found that the receptor of this binding site is α9β1 integrin. In addition, using an antibody for the novel binding motif, we observed that this novel motif is involved in development of anti-type II collagen antibody-induced arthritis.

## Materials and Methods

### Ethics statement

Mice were kept under specific pathogen-free conditions, and provided food and water ad libitum. Every effort was made to minimize suffering during injections, and all surgery was performed in humanely sacrificed mice. All animal experiments were performed in accordance with the guidelines of the Bioscience Committee of Hokkaido University and were approved by the Animal Care and Use Committee of Hokkaido University (Approval license No. 13-0131).

### Reagents

Anti-β1 integrin (HMβ1-1) antibody, anti-β3 integrin (2C9.G2) antibody, anti-α1 integrin (Ha31/8) antibody, anti-α2 integrin (HMα2) antibody, anti-α4 integrin (R1-2) antibody, anti-α5 integrin (HMα5) antibody, anti-αv integrin (RMV-7), and anti-αL integrin (M17/4) were obtained from BD Bioscience. Anti-CD44 (KM81) was obtained from Abcam. Normal hamster IgG, normal rat IgG, and normal rabbit IgG were obtained from Jackson ImmunoResearch. Anti-α9β1 integrin (55A2C) antibody was prepared as described previously [21]. Anti-OPN (O-17) antibody [22], which is a polyclonal antibody for the N-terminal peptide of mouse OPN, was obtained from Immuno-Biological Laboratories. Anti-OPN (C-term), which is an antibody against C-terminal region, and anti-OPN (LRS-EQY) antibody, which is an antibody for the novel cell adhesion motif, were generated from rabbits immunized with synthetic peptide RYLKFRISHELESSSSEVN and LRSKSRSFQVSDEQY, respectively, as previously described [23]. MMP-3 and MMP-7 were obtained from PeproTech and R&D systems.

### Cell cultures

B16-BL6 mouse melanoma cells and NIH3T3 cells expressing mouse α9 integrin (α9/NIH) or mouse α4 integrin (α4/NIH) [21] were cultured in DMEM supplemented with 10% FCS and maintained at 37°C in a humidified atmosphere of 5% CO_2_.

### Enzyme cleavage

Mouse OPN protein was purified from supernatant of mOPN cDNA-transfected CHO cells as previously described [22], Mouse OPN was cleaved by MMP-3 and -7 according as previous report [13]. Briefly, 10 ng of MMP was used with 200 ng of OPN in equal volume of cleavage buffer (200 mM NaCl, 50 mM Tris-HCl, pH 7.6, 5 mM CaCl2) for 15 min at 37°C.

### Western Blotting analysis

OPN or OPN treated with MMPs were fractionated by SDS-PAGE, and transferred to polyvinylidene difluoride membrane (PerkinElmer, Boston, MA). The filters were then immunoblotted with various anti-OPN antibodies. Immunoreactive proteins were visualized using an ECL detection system (Millipore, Bedford, MA).

### Peptide preparation

All peptides used in this study were purchased from Sigma Aldrich Japan (Hokkaido, Japan), and these were synthesized on an automated Syro II peptide synthesizer (MultiSynTech, Witten, Germany), followed by purification with C18 reversed-phase column chromatography. Cysteine residues were introduced at the C-terminal end of each peptide to conjugate with bovine serum albumin (BSA). In case of SVVYGLR, GRGDS, and GDSLAYGLR, cysteine residues were at the N-terminal end for BSA conjugation.

### Cell adhesion assay

Ninety-six-well plates were pre-coated with recombinant OPN in the absence or presence of MMP-3/7, or synthesized OPN peptide as shown in the results section at 37°C for 1 h, followed by treatment with 0.5% BSA in PBS for 1 h at room temperature. Cells in the presence or absence of various antibodies for integrin, CD44 or OPN were suspended in DMEM containing 0.25% BSA and 200 µL of cell suspension (at a cell density of 5×10^4^ cells/well) were applied to 96-well plates and incubated for 1 h at 37°C. The medium was removed from the plates and all wells were washed twice. The adherent cells were fixed and stained by 0.5% crystal violet in 20% methanol for 30 min. All wells were rinsed three times with water, and adherent cells were lysed with 20% acetic acid. The resulting supernatant from each well was analyzed by an immunoreader (Bio-rad, Rochmond, CA), and the absorbance at 595 nm was measured to determine the relative number of cells adhering to each well.

### Scratch wound assay

Thirty-five millimeter tissue culture dishes were coated with 10 µg/mL of various OPN peptides overnight at 4°C, followed by treatment with 0.5% BSA in PBS for 1 h at room temperature. α9/NIH cells were suspended with trypsin/EDTA and plated onto coated dishes at a density of 1×10^6^ cells/35-mm dish. After 2 h, cell cultures were scratched with a single pass of a pipette tip, and photographed. Cells were incubated at 37°C for 15 h. Cultures were washed twice with PBS, fixed and stained in 0.5% crystal violet in 20% methanol for 30 min, and photographed. Cells that had migrated into wound spaces in six high-powered fields (HPF) per dish were counted. Cell numbers are expressed as the number of migrated cells per six HPF.

### Cell proliferation assay

Cell proliferation assay was performed with Cell Counting Kit-8 (Dojindo, Kumamoto, Japan). 96-well plates were coated with 10 µg/mL of various OPN peptides overnight at 4°C, followed by blocking with 0.5% BSA in PBS for 1 h at room temperature. α9/NIH cells were suspended in DMEM containing 10% FCS and plated in 96-well plates at 2×10^4^ cells/well, then incubated for 2 h at 37°C. The medium was replaced with DMEM/0.25% BSA without FCS after washing once with the same medium and incubated for another 13 h. WST-8 (2-(2-methoxy-4-nitrophenyl)-3-(4-nitrophenyl)-5-(2,4-isulfophen-yl)-2H-tetrazolium, monosodium salt) was then added and absorbance of reduced WST-8 was measured after 2 h the absorbance at 450 nm with a reference wavelength of 620 nm by an immunoreader (Bio-Rad).

### Flow cytometry

For α9β1 integrin expression, B16-BL6 cells were blocked by normal goat serum then sequentially incubated with biotinylated anti-mouse α9β1 integrin (55A2C) antibody, followed by streptavidin-phycoerythrin. Biotinylated hamster IgG was used as a negative control. For CD44 expression, α4/NIH cells or B16-BL6 cells after blocking were incubated with anti-mouse CD44 (KM81) antibody, followed by anti-rat IgG-phycoerythrin. Rat IgG2a was used as a negative control. Analyses were performed on FACSCalibur flow cytometer (BD Biosciences).

### Isolation of ankle joint synovial tissues

The ankle joint portion of a leg was cut and then the dermal, subcutaneous, tendinous, and muscle tissues were surgically taken off from joints under a dissecting microscope. The remaining soft tissues were surgically separated from bone tissues and were used as synovial tissues.

### Real-time PCR

Total RNA from synovial tissues from arthritic joints was extracted using Trizol (Life Technologies) and first-strand cDNA was generated with a first-strand cDNA synthesis kit (GE Healthcare Biosciences). Real-time quantitative PCR was performed using LightCycler FastStart DNA Master SYBR Green I Systems (Roche Diagnostics). The specific primers used were: MMP-3: 5′-ATGAAAATGAAGGGTC-3′ and 5′-ACCAGCTATTGCTCTTC-3′; MMP-7: 5′-ATGCAGCTCACCCTGTT-3′ and 5′-TCACAGCGTGTTCCTCTTTCC-3′. The expression level of mRNA was calculated using the calibration curve method using LightCycler software version 3. Data were standardized to G3PDH.

### Mouse experiments

Arthritis was induced using an arthritogenic 4-clone monoclonal antibody cocktail kit containing clone A20, F10-21, D8-6, and D1-2G, which recognize the epitopes on various species of type II collagen. We purchased this kit (Cat#62200) from IBL, Gunma, Japan, but it is no longer available from IBL. The same kit (Cat# 62100) is available from Chondrex Inc, Redmond, WA. Now, Chondrex Inc developed 5-clone cocktail, clone A20, F10-21, D8-6, D1-2G, and D2-112, in order to induce arthritis effectively compared to 4-clone cocktail. Briefly, 7-week-old female BALB/c mice (Charles River, Yokohama, Japan) were injected intravenously with a mixture of four anti-type II collagen monoclonal antibodies (2 mg each) on day −3, followed by an intraperitoneal injection of 50 µg of LPS (0111:B4) on day 0 as reported previously [15]. Anti-OPN (LRS-EQY) antibody was administered intraperitoneally at doses of 400 µg per mouse on days −4 and 0. The clinical severity of arthritis was graded up to 9 days after LPS administration in each of the four paws on a 0–4 scale. The disease severity was recorded for each limb as follows: 0, normal; 1, focal slight swelling and/or redness in one digit; 2, moderate swelling and erythema of >2 digits; 3, marked swelling and erythema of the limb; and 4, maximal swelling, erythema, deformity, and/or ankylosis. Mice were scored in a double-blind manner.

### Histology

Histological assessment of the arthritic joints on day 14 was carried out using sections stained with fast green/Safranin-O or hematoxylin and eosin as described previously [21].

### Statistical analysis

Data are presented as means ±SEM and are representative of at least three independent experiments. The statistical significance of differences between groups was calculated with a Student's t test for single comparisons and one-way ANOVA followed by Student's t test with Bonferroni's correction for multiple comparisons. Differences were considered to be significant when *P*<0.05 (*) or 0.005 (**). The analysis was performed with the statistical software JMP11 for Windows (SAS Institute Inc).

## Results

### A C-terminal region of MMP-3/7-cleaved OPN binds to α9β1 integrin

**Figure 1:**
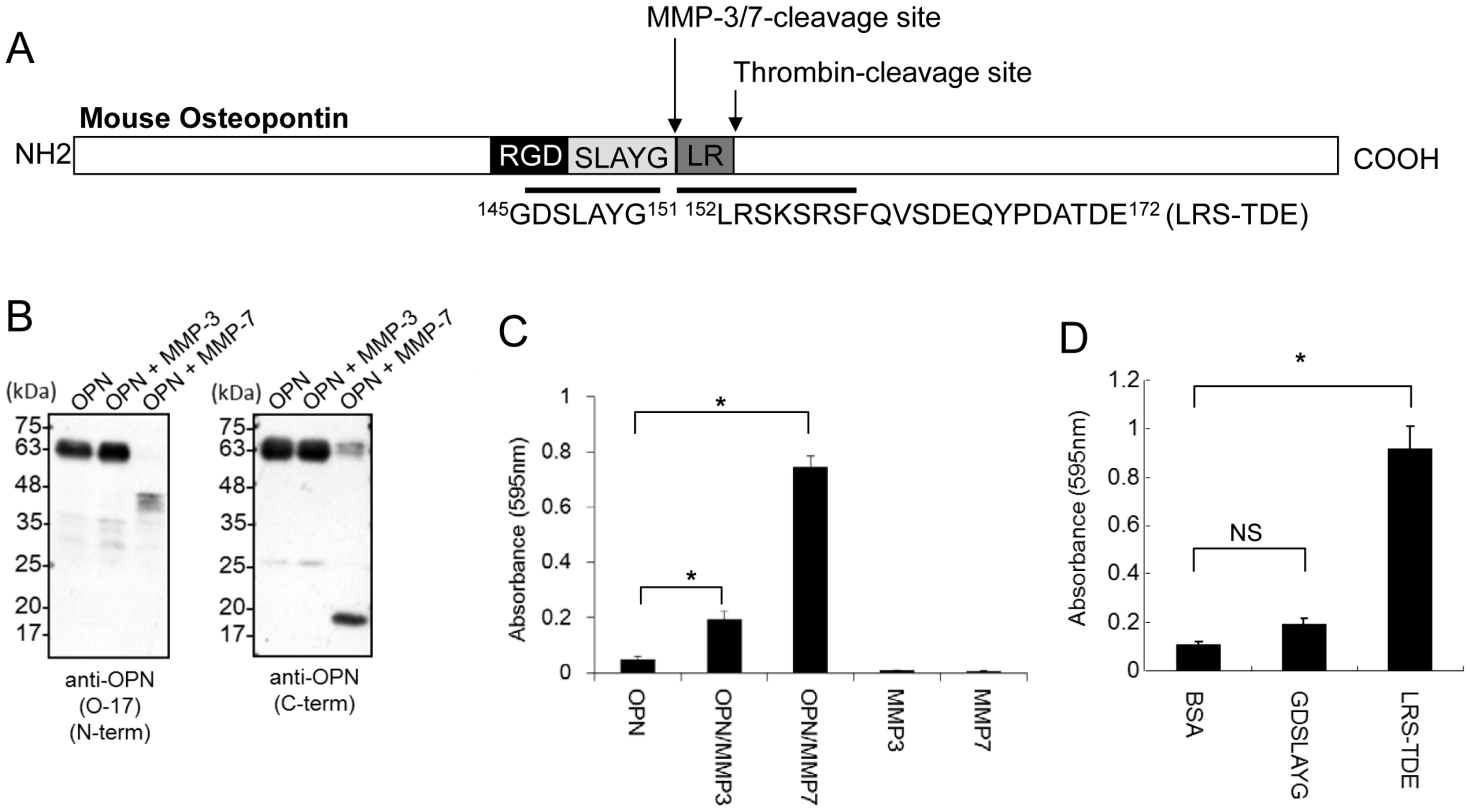
A novel binding region, LRS-TDE, binds to B16-BL6 cells. A. The positions of MMP-3/7- and thrombin-cleavage sites (between G^151^ and L^152^, or R^153^ and S^154^, respectively) and the peptides used for the cell adhesion test are indicated. B. Western blot analysis of OPN or OPN treated with MMP-3 or MMP-7 using anti-OPN antibodies. C. Cell adhesion test using OPN (2 µg/mL) in the absence or presence of MMP-3 or -7, or PBS in the presence of MMP-3 or -7 to B16-BL6 mouse melanoma cells. *P<0.05 versus OPN only, one-way ANOVA followed by Student's t test with Bonferroni's correction. Data are presented as means ±SEM from three independent experiments. D. Cell adhesion test using GDSLAYG and LRS-TDE peptides to B16-BL6 mouse melanoma cells. *P<0.05 versus BSA, one-way ANOVA followed by Student's t test with Bonferroni's correction. Data are presented as means ±SEM from three independent experiments.

OPN can be cleaved by MMP-3/7 between G^151^ and L^152^ and by thrombin between R^153^ and S^154^ [13]. The MMP-3/7 and thrombin cleavage sites in OPN are shown Fig. 1A. To confirm mouse OPN can be cleaved by MMP-3 or -7, we performed western blot analysis using antibody against N-terminal or C-terminal sequences of OPN. Then, we detected cleaved fragments of OPN by MMP-7, but hardly detected cleaved form by MMP-3 (Fig. 1B). To determine whether MMP-cleaved OPN has cell adhesion ability, we used a B16-BL6 mouse melanoma cell adhesion assay with OPN or OPN treated with MMP-3 or -7. Interestingly, binding effect of B16-BL6 cells to plate coated with MMP-3 as well as MMP-7 is enhanced (Fig. 1C). This result suggests that MMP-3 affects OPN function weakly. Thus, mouse OPN, like human and rat OPN, is a substrate for MMP-7 and MMP-3. Next, we asked whether cleavage site by MMP is involved in cell adhesion. For this experiment, we synthesized ^145^GDSLAYG^151^ and ^152^LRSKSRSFQVSDEQYPDATDE^172^ peptides, (referred to as LRS-TDE peptide, hereafter) which are peptides mimicking the C-terminal end in the N-terminal fragment or the N-terminal end in the C-terminal fragment of MMP-cleaved mouse OPN, respectively. We found that LRS-TDE peptide, but not GRSLAYG showed strong cell adhesion to B16-BL6 cells (Fig. 1D).

**Figure 2:**
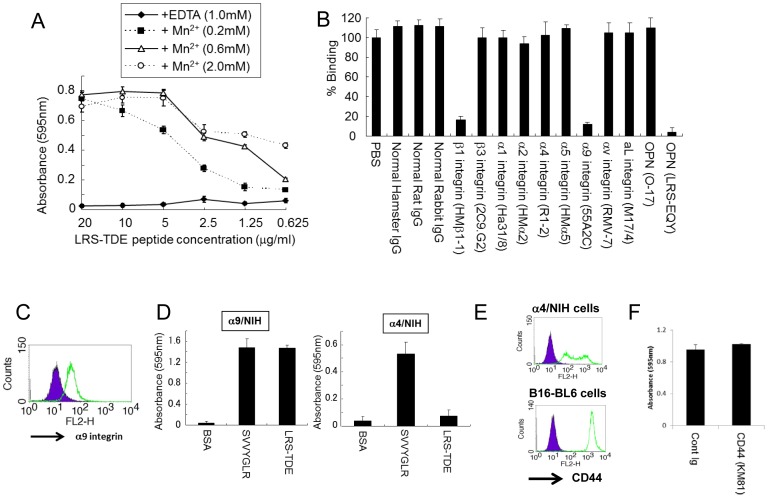
LRS-TDE peptide binds to α9β1 integrin. A. Binding of LRS-TDE peptide to B16-BL6 cells in the presence of Mn^2+^ ions or EDTA. B16-BL6 cells were plated onto dishes coated with LRS-TDE peptide as described in Materials and Methods. B. Binding of LRS-TDE peptide (5 µg/mL) to B16-BL6 cells in the presence of antibodies (10 µg/mL) for various integrins or OPN. C. Surface expression of α9β1 integrin on B16-BL6 cells. Shaded peak represents cells stained with control antibody, and open peak represents cells stained with anti-α9β1 integrin (55A2C). D. Binding of SVVYGLR (5 µg/mL) or LRS-TDE peptide (5 µg/mL) to NIH3T3 cells expressing mouse α9 integrin (α9/NIH) or mouse α4 integrin (α4/NIH). E. Surface expression of CD44 on α4/NIH cells or B16-BL6 cells. Shaded peak represents cells stained with control antibody (rat IgG2a), and open peak represents cells stained with anti-CD44 (KM81). F. Binding of LRS-TDE peptide to B16-BL6 cells in the presence of antibodies (20 µg/mL) for CD44 or control Ig (rat IgG2a).

We then tried to identify the cell surface receptor on B16-BL6 cells binding with LRS-TDE peptide. We hypothesized that receptor(s) should be an integrin because the main OPN receptors are integrins. Integrins require divalent cations for activity [24]–[26]. Therefore, we tested the effect of divalent cations in cell adhesion assay by using EDTA used for depletion of cations. We found that the cell binding was completely inhibited by EDTA, but augmented along with the dose of exogenously added divalent cations (Fig. 2A), indicating that the receptor on B16-BL6 cells for LRS-TDE peptide is indeed an integrin.

To identify the integrin receptor, the inhibitory effects of various antibodies against integrins were examined in cell adhesion assay. As shown in Fig. 2B, the adhesion of LRS-TDE peptide to B16-BL6 cells was inhibited by specific antibodies against α9β1 integrin and β1 integrin. Surface expression of α9β1 integrin on B16-BL6 cells are analyzed by flow cytometer (Fig. 2C). We therefore concluded that the receptor for LRS-TDE peptide is α9β1 integrin.

To confirm specific binding of LRS-TDE peptide with mouse α9β1 integrin, a cell adhesion assay was performed using NIH3T3 cells expressing α9 integrin (α9/NIH) or α4 integrin (α4/NIH). SVVYGLR peptide was used as the positive control for binding to α9 integrin and α4 integrin, because the SVVYGLR sequence in OPN can bind both integrins [4], [27]. We found that LRS-TDE peptide binds to α9/NIH cells but not α4/NIH (Fig. 2D). Nevertheless, both α9/NIH and α4/NIH cells bind to SVVYGLR, as expected. Previous report indicates that C-terminal domain of OPN interacts with CD44 [28]. Therefore, CD44 is one of candidate receptor on B16-BL6 for the binding of LRS-TDE peptide. Flow cytometric analysis revealed CD44 expression on B16-BL6 cells (Fig. 2E). However, we found the interaction between LRS-TDE peptide and B16-BL6 cells was not inhibited by anti-CD44 (KM81) antibody (Fig. 2F), which is the neutralizing antibody for chemotaxis between C-terminal domain of OPN and CD44 [28]. It is noted that LRS-TDE peptide does not bind to α4/NIH cells, despite expressing CD44 on the cells (Fig. 2E). These results indicate that the receptor of LRS-TDE peptide is α9 integrin, but not CD44.

### LRS-TDE peptide promotes α9β1 integrin-mediated in vitro wound healing

**Figure 3:**
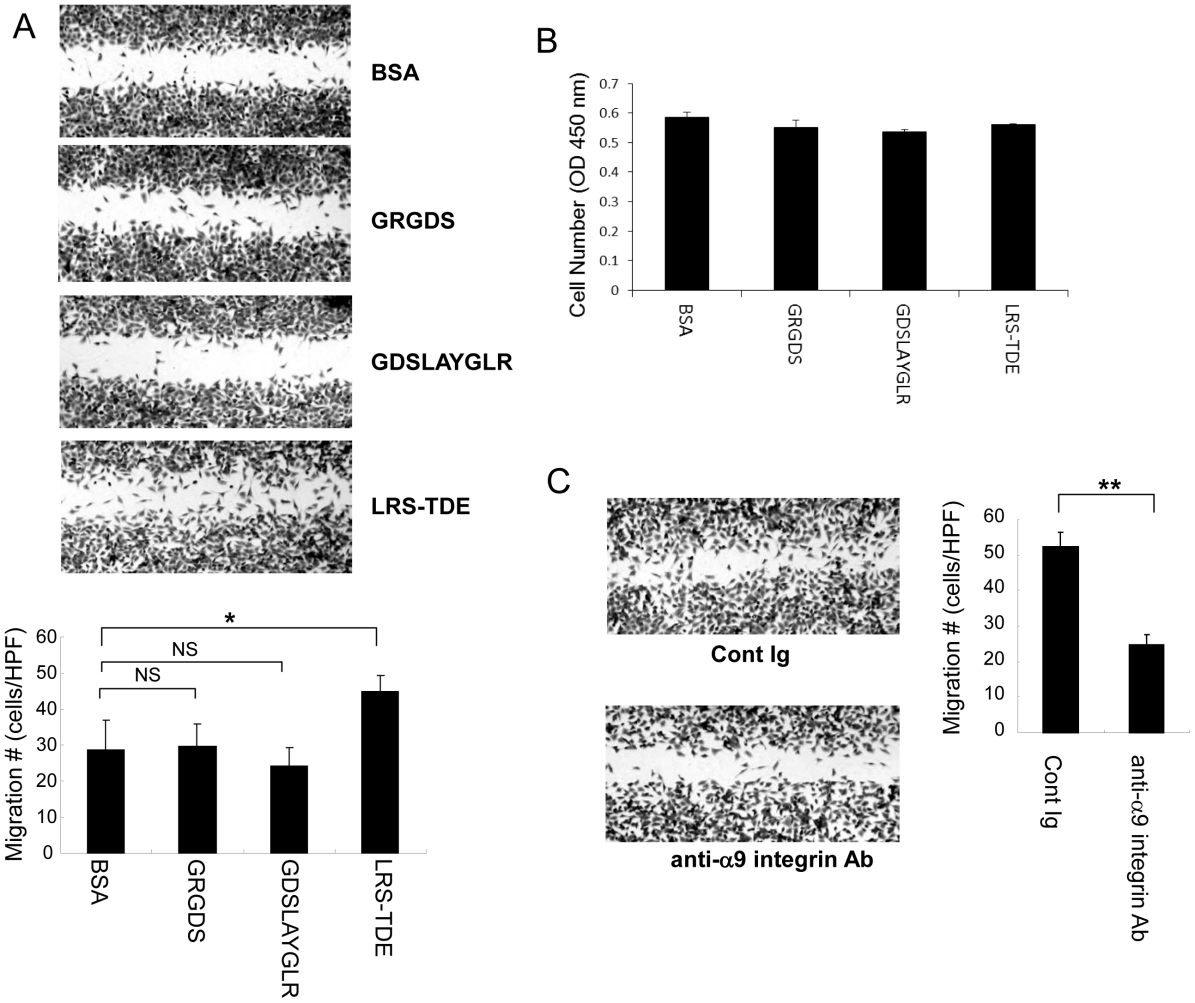
LRS-TDE peptide enhances α9β1 integrin-mediated cell migration. A. α9/NIH cells were plated onto dishes coated with GRGDS, GDSLAYGLR, or LRS-TDE peptides (10 µg/mL), scratched with the tip of a pipette, and allowed to migrate into wound spaces for 15 h. *P<0.05 versus BSA, one-way ANOVA followed by Student's t test with Bonferroni's correction. Data are presented as means ±SEM from three independent experiments. B. Effect of GRGDS, GDSLAYGLR, or LRS-TDE peptides (10 µg/mL) on α9/NIH cells proliferation assessed by the colorimetric assay (WST-8) for the quantification of cell proliferation. C. α9/NIH cells were incubated with control Ig (Hamster IgG) or anti-α9β1 integrin (55A2C) antibody (10 µg/mL). Cells that had migrated into the scratch area were counted. ^**^ *P*<0.005 versus control Ig, Student's t test. Data are presented as means ±SEM from three independent experiments.

We tested the effect of LRS-TDE peptide in a scratch wound healing assay. Scratch wound closure after 15 h was quantified by phase-contrast microscopy. Cells on LRS-TDE peptide showed significantly stronger scratch wound closure than cells on BSA, GRGDS, or GDSLAYGLR (Fig. 3A). The study of cell proliferation revealed that LRS-TDE peptide did not affect α9/NIH cell growth (Fig. 3B), indicating that promoted wound closure on LRS-TDE peptide was not caused by cell growth. We then tested the blocking effect of anti-α9β1 integrin antibody in scratch wound closer on LRS-TDE peptide. Anti-α9β1 integrin antibody almost completely canceled scratch wound healing on LRS-TDE peptides (Fig. 3C). We therefore concluded that a novel binding region, LRS-TDE, is involved in α9β1 integrin-mediated cell migration and cell adhesion, as reflected in scratch wound healing assay.

### The essential requirement of peptide sequence within LRS-TDE peptide for binding to α9β1 integrin

**Figure 4:**
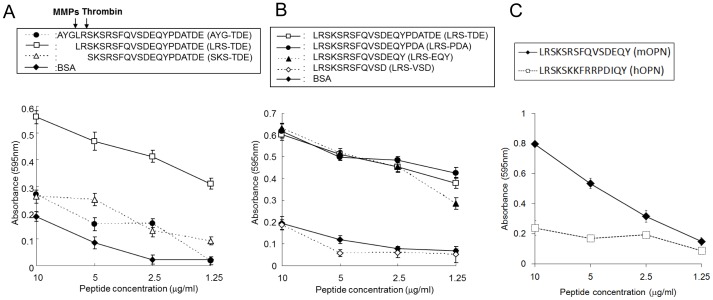
A novel binding region requires MMP-3/7 cleavage to bind to α9β1 integrin. Binding of peptides of different lengths in the N-terminus (A) or C-terminus (B) to B16-BL6 cells. BSA was used as the negative control. A. AYG-TDE peptide mimics full-length OPN. LRS-TDE or SKS-TDE peptides mimic the N-terminus region in the C-terminal fragment of MMP-3/7-, or thrombin-cleaved OPN, respectively. B. Four peptides of different lengths towards the C-terminal were used for cell adhesion to determine binding motif in the LRS-TDE sequence. C. Cell binding effect of LRSKSKKFRRPDIQY peptides, a homologous sequence in human OPN of LRS-TDE peptide, to α9/NIH cells.

To determine whether OPN cleavage by MMP-3/7 is required to bind α9β1 integrin, we synthesized three different peptides as follows. ^149^AYG-TDE^172^ peptide, which contains the MMP-3/7 and thrombin cleavage sites, and is designed to mimic full length OPN. ^152^LRS-TDE^172^ peptide starts at ^152^L, where the MMP-3/7 cleavage site is, and is designed to have the N-terminal end in the C-terminal fragment of MMP-3/7-cleaved OPN. ^154^SKS-TDE^172^ peptide starts at ^154^S, where the thrombin-cleavage site is, and is designed to have the N-terminal end in the C-terminal fragment of thrombin cleaved OPN. We found that LRS-TDE peptide, but not AYG-TDE and SKS-TDE binds to α9/NIH cells (Fig. 4A), suggesting that MMP-3/7 cleavage is critical for creating the peptide, which is recognized by α9β1 integrin.

We then prepared another three different peptides in which some of peptides were deleted in the C-terminal end of LRS-TDE peptide. We found that ^152^LRS-TDE^172^, ^152^LRS-PDA^169^, and ^152^LRS-EQY^166^ peptide bind to α9β1 integrin, but not ^152^LRS-VSD^163^ peptide (Fig. 4B). We therefore concluded that LRSKSRSFQVSDEQY (LRS-EQY) in the C-terminal fragment of MMP-3/7-cleaved OPN is a binding motif for α9β1 integrin. Next, we asked whether the same region of human OPN is involved in the binding to α9 integrin. To answer this question, the homologous 15 aa peptide in human OPN, LRSKSKKFRRPDIQY peptide, was synthesized and cell adhesion assay was performed. Then, we found that LRSKSKKFRRPDIQY peptide does not bind to α9 integrin (Fig. 4C).

### Attenuation of collagen antibody-induced arthritis (CAIA) by administration of anti-LRS-EQY antibody

We previously reported that α9β1 integrin is critical for the development of CAIA [21]. We now considered whether the interaction between LRS-EQY motif in MMP-3/7-cleaved OPN and α9β1 integrin is involved in the development of CAIA. The mouse model of inflammatory arthritis was induced by intravenous administration of a mixture of four anti-type II collagen monoclonal antibodies followed by intraperitoneal administration of LPS. We have also shown that up-regulated OPN expression is critically involved in the development of this murine arthritis model [15].

**Figure 5:**
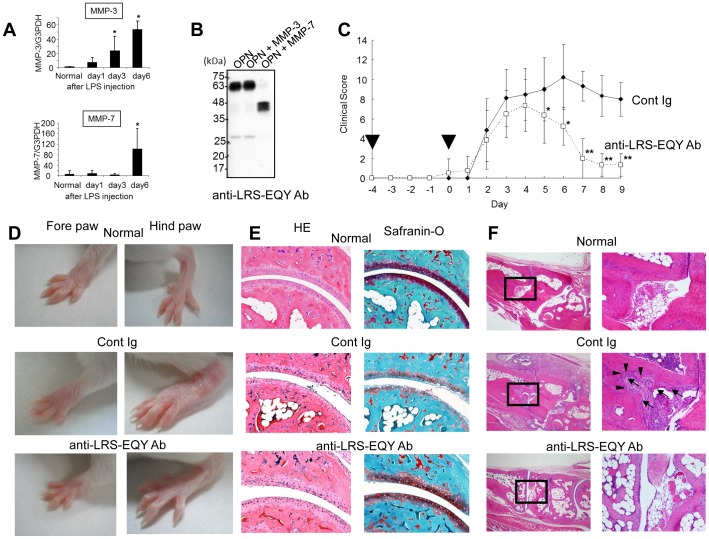
Treatment with an antibody for LRS-EQY protects mice from inflammatory arthritis. A. Kinetic analysis of gene expression of MMP-3 or MMP-7. Total RNA extracted from ankle joint synovial tissues of individual mice with arthritis was subjected to quantitative real-time PCR. Expression levels are normalized to G3PDH (n = 4 per group). *P<0.05 versus normal, one-way ANOVA followed by Student's t test with Bonferroni's correction. Data are presented as means ±SEM from three independent experiments. B. Western blot analysis of OPN or OPN treated with MMP-3 or MMP-7 using anti-LRS-EQY Ab. C. Arthritis scores of arthritic mice treated with control Ig (rabbit IgG) or an anti-LRS-EQY antibody at the indicated time points (*arrow heads*) (n = 8 per group). *P<0.05, ^**^ *P*<0.005 versus control Ig, Student's t test. Data are presented as means ±SEM from three independent experiments. D. Representative images of gross appearances of the forepaw and hindpaw on day 8 are shown. E, F. Representative histology of normal joints and arthritic joints on day 14 from mice treated with control Ig (normal rabbit IgG) or an anti-LRS-EQY antibody. Sections were stained with hematoxylin and eosin or Safranin-O. F. Boxed areas in left panels are magnified in right panels. *Arrowheads* or *arrows* indicate pannus formation or bone erosion, respectively. *P<0.05, ^**^ *P*<0.005 versus control Ig, Student's t test. Data are presented as means ±SEM from three independent experiments.

We first analyzed MMP-3 and MMP-7 expression in synovial tissues of arthritic joints by real-time polymerase chain reaction (PCR). Gene expression of MMP-3 was significantly up-regulated on day 3 and day 6 after LPS injection. MMP-7 expression was induced on day 6 (Fig. 5A). This result suggests that MMP-cleaved OPN may be induced after 3 days in arthritic joints. Next, we generated an antibody against LRS-EQY peptide, anti-LRS-EQY antibody, which successfully detected MMP-cleaved OPN as well as full length OPN (Fig. 5B). It should be noted that an anti-LRS-EQY antibody can inhibit the adhesion of LRS-TDE peptide to α9β1 integrin (Fig. 2B). Using this antibody, we tested the arthritis blocking effect. Mice were treated twice with an anti-LRS-EQY antibody before the onset of arthritis on days −4 and 0. Joint swelling in control Ig-treated mice became evident on day 2 and reached a peak on day 6, whereas the severity of arthritis was significantly reduced by the anti-LRS-EQY antibody after day 5 (Fig. 5C). Fig. 5D is a representative image of the gross appearances of an arthritic joint treated with an anti-LRS-EQY antibody on day 8. In concert with the clinical score, histological analysis demonstrated marked differences between anti-LRS-EQY antibody-treated and control Ig-treated mice (Fig. 5E). Cartilage destruction was assessed by Safranin-O staining. As shown in Fig. 5E, faint Safranin-O staining was detected in the arthritic joints of control Ig-treated mice on day 14 after LPS injection, indicating that cartilage destruction was present. In contrast, Safranin-O staining of articular cartilage in anti-LRS-EQY antibody-treated mice was similar to that of normal mice (Fig. 5E), indicating that articular cartilage was well preserved in anti-LRS-EQY antibody-treated mice. We also detected remarkable proliferation of synovial cells and pannus formation at the base of the metatarsal bones in control Ig-treated mice (Fig. 5F, *arrowhead*). Furthermore, an irregular bone surface was apparent in the arthritic joints of control Ig-treated mice, which indicated intensive bone erosion (Fig. 5F, *arrow*). The appearance of these pathological features of CAIA clearly diminished with administration of an anti-LRS-EQY antibody.

## Discussion

Osteopontin can be cleaved by thrombin [9] and MMP-3 (stromelysin-1) or -7 (matrilysin) [13] at positions ^153^R/S^154^ and ^151^G/L^152^, respectively. A cleavage site for MMP-3/7 resides just two amino acid residues towards the N-terminal from the thrombin-cleavage site. Thrombin-cleavage of OPN generates a N-terminal fragment that exposes a cryptic adhesive motif, SVVYGLR, on its C-terminal end, which has been reported to be recognized by both α4 integrin [29] and α9β1 integrin [4]. In contrast, the N-terminal fragment cleaved by MMP-3/7 does not reveal an additional α9β1 integrin binding site [7], [13], but not α4 integrin [7], [30].

The cell binding motif in the C-terminal fragment of thrombin- or MMP-3/7-cleaved OPN has not yet been identified. In this study using B16-BL6 mouse melanoma cells, we found that LRSKSRSFQVSDEQYPDATDE (LRS-TDE) peptide in the C-terminal fragment of OPN cleaved by MMP-3/7 binds to α9β1 integrin (Fig. 2D). A cell adhesion assay using a synthesized peptide showed that MMP-3/7 cleavage of OPN leads to exposure of novel α9β1 integrin-binding motif, LRSKSRSFQVSDEQY (Fig. 4A).

In a previous study, we found that α9β1 integrin is critical in the development of CAIA [21]. In the current study, we used an antibody for LRS-EQY peptide (anti-LRS-EQY) and found that the novel interaction is involved in inflammatory arthritis. Real-time PCR analysis showed that MMP-3 and MMP-7 mRNA in ankle joints were up-regulated on day 3 and day 6 after LPS injection, respectively (Fig. 5A), suggesting that MMP-3/7-cleaved OPN is not generated until at least day 3. In an *in vivo* experiment, we observed that anti-LRS-EQY antibody significantly protected mice from inflammatory arthritis after 5 days (Fig. 5C). This 5th day, when the antibody is functional, is consistent with the induction of MMP-3/7-cleaved OPN.

We reported that treatment with antibodies for SLAYGLR (M5 Ab) [15] and α9β1 integrin [21] delayed the onset of arthritis as well as attenuating its severity, suggesting that the interaction between thrombin-cleaved OPN and α9β1 integrin is important for the development of arthritis in the early phase. However, treatment with anti-α9β1 integrin antibody reduced joint swelling to a normal state after reaching its peak on day 3 (clinical score of 2), whereas treatment with M5 Ab exacerbated the arthritis gradually after onset, suggesting that perhaps other α9β1 integrin ligands are involved in the late phase of arthritis. Treatment with an anti-LRS-EQY antibody attenuated the severity of arthritis from day 5 after LPS injection. This strongly suggests the critical involvement of the interaction between α9β1 integrin and MMP-3/7-cleaved OPN via a LRS-EQY motif in the development of inflammatory arthritis in the late phase.

OPN undergoes post-translational modification by transglutaminase 2 (TG2), which is a protein cross-linking enzyme, as well as thrombin and MMP-3/7, and OPN is polymerized by TG2. These modifications provide a new function for OPN, which is binding to α9β1 integrin. It is conceivable that polymeric OPN may be involved in inflammatory arthritis since TG2 is up-regulated in inflammatory diseases [31]. The generation of antibody against polymeric OPN to inhibit the interaction between polymeric OPN and α9β1 integrin may be useful. Further research is required to understand how polymeric OPN is involved in inflammatory arthritis.

The LRSKSRSFQVSDEQY novel binding motif is within the mouse OPN sequence. The homologous sequence in human OPN is LRSKSKKFRRPDIQY (underlines indicate mismatch amino acids compared with mouse OPN). We tested whether this human homolog peptide has a binding ability to human α9β1 integrin using a cell adhesion test, but we were not able to conclude that the peptide bound to α9β1 integrin (Fig. 4C). However, we cannot exclude the possibility that the longer homolog peptide directed to the C-terminal in the C-terminal fragment of MMP-cleaved human OPN binds to α9β1 integrin.

In conclusion, we identified a novel interaction between the LRS-EQY motif in the C-terminal fragment of mouse OPN cleaved by MMP-3/7 and α9β1 integrin, and provide evidence for a potential role of this novel binding motif in the regulation of α9β1 integrin-mediated cell adhesion and migration and in the pathogenesis of inflammatory arthritis. Future studies will be directed at understanding the mechanisms by which LRS-EQY motif and its interactions with α9β1 integrins affect other cellular functions.
